# Niche overlap between a cold-water coral and an associated sponge for isotopically-enriched particulate food sources

**DOI:** 10.1371/journal.pone.0194659

**Published:** 2018-03-26

**Authors:** Dick van Oevelen, Christina E. Mueller, Tomas Lundälv, Fleur C. van Duyl, Jasper M. de Goeij, Jack J. Middelburg

**Affiliations:** 1 Department of Estuarine and Delta Systems, Royal Netherlands Institute for Sea Research (NIOZ-Yerseke) and Utrecht University, Yerseke, The Netherlands; 2 Sven Lovén Centre for Marine Sciences, Tjärnö, University of Gothenburg, Strömstad, Sweden; 3 Department of Marine Microbiology and Biogeochemistry, Royal Netherlands Institute for Sea Research (NIOZ-Texel) and Utrecht University, Texel, The Netherlands; 4 Department of Freshwater and Marine Ecology, Institute for Biodiversity and Ecosystem Dynamics, University of Amsterdam, The Netherlands; 5 Utrecht University, Department of Earth Sciences, Utrecht, The Netherlands; Auckland University of Technology, NEW ZEALAND

## Abstract

The cold-water coral *Lophelia pertusa* is an ecosystem engineer that builds reef structures on the seafloor. The interaction of the reef topography with hydrodynamics is known to enhance the supply of suspended food sources to the reef communities. However, the reef framework is also a substrate for other organisms that may compete for the very same suspended food sources. Here, we used the passive suspension feeder *Lophelia pertusa* and the active suspension feeding sponge *Hymedesmia coriacea* as model organisms to study niche overlap using isotopically-enriched algae and bacteria as suspended food sources. The coral and the sponge were fed with a combination of ^13^C-enriched bacteria/^15^N-enriched algae or ^15^N-enriched bacteria/^13^C-enriched algae, which was subsequently traced into bulk tissue, coral skeleton and dissolved inorganic carbon (i.e. respiration). Both the coral and the sponge assimilated and respired the suspended bacteria and algae, indicating niche overlap between these species. The assimilation rates of C and N into bulk tissue of specimens incubated separately were not significantly different from assimilation rates during incubations with co-occurring corals and sponges. Hence, no evidence for exploitative resource competition was found, but this is likely due to the saturating experimental food concentration that was used. We do not rule out that exploitative competition occurs in nature during periods of low food concentrations. Food assimilation and respiration rates of the sponge were almost an order of magnitude higher than those of the cold-water coral. We hypothesize that the active suspension feeding mode of the sponge explains the observed differences in resource uptake as opposed to the passive suspension feeding mode of the cold-water coral. These feeding mode differences may set constraints on suitable habitats for cold-water corals and sponges in their natural habitats.

## Introduction

Sessile suspension feeders extract organic resources from the water column and contribute significantly to the cycling of organic matter and inorganic nutrients in many marine ecosystems [1–4]. Being non-mobile, the growth of sessile suspension feeders is mostly limited by availability of space and food in the ambient water column [5]. Some suspension feeders, such as calcifying corals, significantly modify the topography of the seafloor by forming large reef structures. The interaction of the reef topography with hydrodynamics is known to enhance the supply of limiting resources to the reef communities making them archetypical examples of ecosystem engineers [6]. Yet, these structures may also be used as habitat by other suspension feeders that in turn may compete with the ecosystem engineer for the very same suspended resources.

Cold-water corals are passive suspension feeders that grow in the deep sea, where organic resources are scarce due to degradation of surface-produced organic matter during the downward transit to the seafloor [7]. *Lophelia pertusa* (Linnaeus, 1758) is the dominant reef-building cold-water coral species in the North Atlantic Ocean [8] that forms reef structures of several meters to hundreds of meters in height [9–11]. These structures interact with the (tidal) hydrodynamics [12, 13] in a way that the flux of limiting organic resources to the reef communities is temporarily enhanced [14–16]. Moreover, field and laboratory observations indicate that *L*. *pertusa* feeds on a broad range of different food sources ranging from dissolved to particulate suspended organic matter [17–20], which further increases its capacity to utilize resources under limiting conditions.

The carbonate reef framework is also an important site for settlement, refuge and feeding ground for many associated organisms [21]. A dominant taxonomic group of associated organisms in cold-water coral reef communities are sponges [22, 23]. Sponges are active suspension feeders with a high filtering capacity that allows them to extract food particles from the water column [24]. The food spectrum of deep-water sponges is broad [25–27] and thereby overlaps, at least partially, with that of cold-water corals. Habitat formation provided by the cold-water coral reef framework may therefore lead to exploitative competition for limiting suspended resources between cold-water corals and associates sponges if they share suspended resources.

*Hymedesmia coriacea* (Fristedt, 1885) is a sponge species that is frequently found on Atlantic cold-water coral reefs [28], where it often lives in close contact with living *L*. *pertusa* and sometimes overgrows the coral [29]. We used *L*. *pertusa* and *H*. *coriacea* as coral–sponge couple to study resource competition using isotopically-labelled algae and bacteria as suspended food sources. The coral *L*. *pertusa* and the sponge *H*. *coriacea* were fed with a combination of ^13^C-enriched bacteria/^15^N-enriched algae or ^15^N-enriched bacteria/^13^C-enriched algae, both separately and placed together. Food assimilation into tissue and coral skeleton, and respiration was used to investigate resource uptake and metabolic activity of the deep-water coral and sponge. A comparison of food processing rates of separate coral and sponge specimens versus the coral-sponge couple allowed identifying whether niche overlap occurred.

## Materials and methods

The experiments described below follow a comparable approach as in Mueller et al. [30], Middelburg et al. [31] and in particular Van Oevelen et al. [32]. The data of the ‘coral only’ experiments in the present manuscript are identical to those described in Van Oevelen et al. [32], but are here presented in different units to facilitate comparison with the associated sponge.

### Sampling location and maintenance

Corals and sponge specimens were collected from the Tisler Reef under permission granted by the Norwegian Directorate of Fishery. The Tisler Reef is located east of the Tisler islands in the sill area of the Koster fjord that connects to the Skagerrak at the border between Norway and Sweden [10]. The living parts of the reef cover a large part of the sill and extend over an area of approximately 1,200 × 200 m at a water depth of 70 to 155 m [10]. The flow direction over the sill fluctuates irregularly between NW and SE with current velocities that are generally between 0 to 50 cm s^-1^ but can reach peaks in excess of 70 cm s^-1^ [33]. The water temperature generally varies between 6 to 9 °C throughout the year [10], but occasionally higher temperatures (exceeding 12 °C) have been observed [34]. The particulate organic carbon (POC) concentration in the benthic boundary layer varies between 43.5 to 106.3 μg POC L^-1^ and depositional fluxes of POC ranges from 18–1485 mg POC m^−2^ d^−1^ with an annual average of 459 mg POC m^−2^ d^−1^ [33].

Both species were collected from a water depth of approximately 110 m using the remotely operated vehicle Sperre Subfighter 7500 DC. Specimens were placed in cooling boxes filled beforehand with cold seawater (7–8 °C) and transported within a few hours to the laboratory at the Sven Lovén Centre in Tjärnö (Sweden). After arrival, coral samples were clipped to approximately the same size and living coral polyps from the reef branches with encrusting sponge were removed to prepare sponge specimens. All specimens were maintained in aquaria (~20 L) placed in a dark thermo-constant room (7 °C) for up to 6 wk before used in the experiment as acclimation time. Sand-filtered (1–2 mm particle size) bottom water from 45 m depth out of the adjacent Koster fjord (salinity 31) was continuously flushed through the aquaria (~1 L min^-1^). From experience at the station, it is known that the sand-filtered water still contains sufficient organic particles so that no extra food had to be provided during the acclimation period.

### Preparation of labeled food substrates

The labelled food sources used in this experiment are identical to those described in Van Oevelen et al. [32]. Bacteria (~1 μm) were cultured by adding a few mL of natural seawater from the Oosterschelde estuary (Netherlands) to M63 culture medium adjusted after Miller [35]. In the medium either 50% of glucose (3 g L^-1^) or 50% of NH_4_Cl (1.125 g L^-1^) was replaced by its heavy isotope equivalent (Cambridge Isotopes, 99% ^13^C, 99% ^15^N) to obtain ^13^C or ^15^N isotopically-enriched bacteria. After 3 d of culturing in the dark, bacteria were concentrated by centrifugation (14500 × g), after which the concentrate was rinsed 3 times with 0.2-μm filtered sea water to remove residual label and stored frozen (-20 °C) until further use.

Single (^13^C or ^15^N) isotopically-enriched diatoms were cultured axenically in F/2 culture medium adjusted after Guillard [36]. In this medium, either 80% of the NaHCO_3_ or 70% of the NaNO_3_ was replaced by its heavy isotope equivalent (Cambridge Isotopes, 99% ^13^C, 99% ^15^N). The medium was injected with a sterile culture inoculum of the diatom *Thalassiosira pseudonana* (Hasle & Heimdal, 1970) (~5 μm). After 3 wk of culturing with a 12 h light–dark cycle (at a cell density of around 3–4 × 10^6^ cells mL^-1^), diatoms were concentrated by centrifugation at 450 × g. The concentrate was rinsed three times with 0.2-μm filtered seawater to remove residual label and kept at -20 °C until further use.

### Experimental set up and procedure

Prior to the start of the experiment, circular Plexiglas incubation chambers (10 L) were filled with 5-μm filtered Koster fjord bottom water and placed in a thermo-controlled room (7 °C) at the Tjärnö Marine Laboratory. A coral specimen, i.e. a sponge-free *Lophelia pertusa* fragment (10.35 ± 1.31 g DW fragment^-1^, 16.83 ± 2.79 polyps fragment^-1^, mean ± sd, *n* = 6) and a sponge specimen, i.e. dead coral branches overgrown by the encrusting sponge *H*. *coriacea* (0.03 ± 0.01 g DW sponge^-1^, mean ± sd, *n* = 12), and were placed separately or together in the incubation chamber (10 L). Subsequent analysis (see below) showed that the coral carbon biomass (10,128 ± 3,459 μmol C, mean ± sd, *n* = 12) in the incubations was substantially higher than the sponge carbon biomass (451 ± 146 μmol C, mean ± sd, *n* = 12). Coral fragments were kept in an upright position by inserting them into elastic silicone tubes that were mounted on an acrylic plate, which was fixed to the chamber base. A steady water circulation maintained by a motor-driven paddle in the upper part of the incubation chamber (speed: 2 rpm).

The corals and sponges in the incubation chambers were exposed to a 10-day feeding period (12 h feeding day^-1^, 12 h flushing day^-1^). A total food pulse of 108 μmol C L^-1^ was given at the beginning of each daily feeding period consisting of ^13^C bacteria/ ^15^N algae or ^13^C algae/ ^15^N bacteria in a diatom:bacteria ratio of 1:3 (Table 1), resulting in a total food pulse of 2,708 μmol C algae and 8,125 μmol C bacteria per chamber. After each feeding period, chambers were flushed with 5-μm filtered Koster fjord bottom water (140 mL min^-1^) for 12 h (flushing period) to avoid accumulation of waste products such as NH_4_^+^ and CO_2_, to remove remaining food particles and to renew the O_2_ supply.

**Table 1:** Experimental design.

Treatment	^13^C-Alg + ^15^N-Bac	^15^N-Alg + ^13^C-Bac
Coral	*n* = 3	*n* = 3
Sponge	*n* = 3	*n* = 3
Coral + Sponge	*n* = 3	*n* = 3

Experimental design and replicates (*n*) used in this study. The coral *L*. *pertusa* and the sponge *H*. *coriacea* were exposed separately and together to a food mixture consisting of ^13^C-labelled algae + ^15^N-labelled bacteria and to a food mixture composed of ^15^N-labelled algae + ^13^C-labelled bacteria.

After the last flushing period, the incubation chambers were closed for 48 h to measure the production of ^13^C dissolved inorganic carbon (^13^C-DIC) as a proxy for respiration. Pilot experiments (C. E. Mueller unpubl. data), prior work [17, 30–32] and literature reports indicate that changes of pH (standard deviation of 0.04 around a mean pH of 7.8 NBS), dissolved oxygen and ammonium concentration [37] in these 48 h are not sufficient to negatively influence coral or sponge physiology. Samples for DIC analysis were taken before (control) and after the respiration incubation. Water was filtered (GF/F) in a 20-mL headspace vial, which was subsequently poisoned with 10 μL HgCl_2_ and closed with an aluminium cap fitted with a rubber septum and stored upside down for further analysis. After the 48-hours closed incubation, the coral and sponge specimens were stored frozen (-20 °C) for later analysis of the incorporation of ^13^C and ^15^N in their tissues and ^13^C incorporation in the calcium carbonate skeleton of the coral.

Corals and sponges for background isotope measurements (controls) were incubated in parallel under ‘acclimatization’ conditions: i.e. without food addition to the sand-filtered seawater (1–2 mm particle size).

### Sample treatment and analysis

Sponge tissue was scraped from the coral surface, homogenized by pestle and weighed. Coral samples were weighed and homogenized by grinding with a ball Mill for 20 s (MM 2000, Retsch, Haan, Germany). 30 mg of ground coral material, 2–3 mg ground sponge material or a subsample of the POM-filter was transferred to pre-combusted silver boats and decalcified by acidification [38] to analyze the organic coral tissue, sponge tissue and POM, respectively. Subsequently, each sample was simultaneously measured for ^13^C and ^15^N using a thermo Electron Flash EA 1112 analyzer (EA) coupled to a Delta V isotope ratio mass spectrometer (IRMS).

A headspace of 3 mL was created in each DIC sample vial by injecting N_2_ gas through the vial septum. Samples were then acidified with 20 μL of concentrated H_3_PO_4_ to transform DIC into CO_2_. When CO_2_ had exchanged with the vial headspace, 10 μL sample of the headspace gas was injected into an elemental analyzer isotope-ratio mass spectrometer (EA-IRMS). Calculations for ^13^C respiration followed the description for ^13^C tissue assimilation.

The incorporation of metabolic-derived ^13^C in coral skeleton was measured following Mueller et al. [30] and Tanaka et al. [39]; 30 mg of each coral sample (i.e. the organic fraction and inorganic skeleton) was transferred to a silver boat and measured on the EA-IRMS for total ^13^C content. The incorporation into the ^13^C in the inorganic skeleton was obtained by subtracting ^13^C assimilation in the organic fraction from the ^13^C incorporation in the total (organic fraction and inorganic skeleton). Calcification based on metabolic-derived inorganic carbon may only be a small amount of the total calcification [~8%, 40], but is used here as proxy for changes in total calcification [30].

The uptake rates of tracer C or N from the enriched substrates have all been normalized to organic C or N biomass (e.g., μmol C_tracer_ mmol C^-1^ d^-1^) to allow direct comparison of the uptake rates between species. These rates are calculated as from the delta notations obtained from the IRMS: δX (‰) = (R_sample_/R_ref_ -1) × 1000, in which X is the element (C or N), R_sample_ is the heavy: light isotope ratio in the sample and R_ref_ is the heavy: light isotope ratio in the reference material (R_ref_ = 0.0111797 for C and R_ref_ = 0.0036765 for N). The atomic fraction of the heavy isotope (F) in a sample is calculated as F = R_sample_/(R_sample_ + 1). The excess (above background) atomic fraction is the difference between the F in an experimental sample and the atomic fraction in a control sample: E = F_sample_−F_control_. The excess incorporation of ^13^C or ^15^N was divided by the atomic enrichment of each specific food source to convert to total C_tracer_ or N_tracer_ incorporation, respectively, and divided by the incubation time to convert to daily rates. Calcification rates are calculated similarly using the δ^13^C (‰) increase in the CaCO_3_ pool.

Respiration rates are inferred from the excess (E) of ^13^C in the DIC pool during the 48-h incubation as: E = F_end_−F_start_. The excess DIC values are multiplied with the DIC pool (i.e., μmol DIC L^-1^) and chamber volume (10 L), and divided by the atomic fraction enrichment of the food source and the incubation time. Respiration are normalized to the total organic C in the incubation chamber (i.e. depending on the incubation; ‘coral organic carbon tissue’, ‘sponge organic carbon tissue’ or ‘coral + sponge organic carbon tissue’).

### Data analysis

A net growth efficiency (NGE) for the coral and the sponge was calculated from the ‘single species’ experiments from the C incorporation rate into tissue and respiration rates as: NGE = Tissue incorporation / (Tissue incorporation + Respiration). Only ‘single species’ experimental data were for this calculation, because total respiration in mixed assemblages cannot be attributed to single species. As discussed below, this NGE calculation ignores other potential losses including cell shedding or dissolved organic carbon release.

Differences in the C and N incorporation rates between the coral and sponge were tested on log_10_-transformed (to meet the normal distribution assumption) data from the ‘single species’ experiments in 2-way ANOVAs, with C or N uptake as dependent variable and species (i.e. coral or sponge) and food source (i.e. bacteria or algae) as dependent factors. To test whether the incorporation of the coral or sponge was affected by the presence of the other species, incorporation rates by the coral and sponge were tested on log_10_-transformed data in 3-way ANOVAs, with C or N uptake as dependent variable and treatment (i.e. coral or sponge ‘only’ versus coral and sponge together), food source (i.e. bacteria or algae) and element (i.e. C or N) as dependent factors. Significant differences in calcification rates were tested for in an ANOVA on log_10_-transformed data with treatment (i.e. coral only and coral with sponge) and food source as dependent factors. Significant differences in respiration rates and the net growth efficiency were tested for with log_10_-transformed data in an ANOVA with treatment (i.e. coral only and sponge only) and food source as dependent factors. Data are presented as mean ± standard deviation. All raw data presented in this paper are made freely downloadable from doi:10.5281/zenodo.1198189.

## Results

### Carbon and nitrogen uptake and stoichiometry

**Fig 1:**
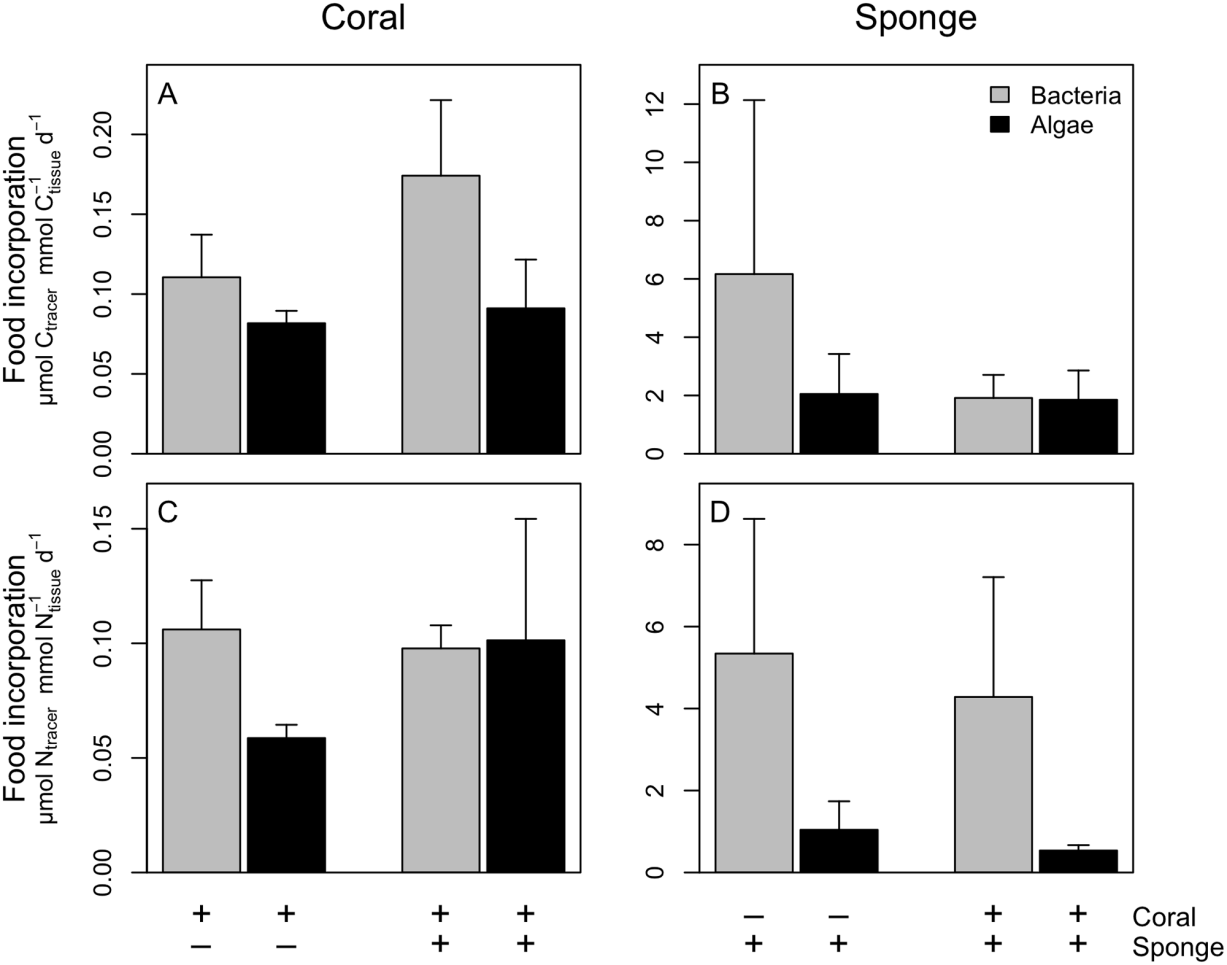
Food incorporation by the coral and sponge. Food incorporation rates, expressed in μmol C_tracer_ mmol C_tissue_^-1^ d^-1^) of algal and bacterial carbon (A, B) and nitrogen (C, D) in the tissue of the cold-water coral *Lophelia pertusa* (A, C) and the sponge *Hymedesmia coriacea* (B, D). The presence (‘+’) or absence (‘-‘) of a species is indicated below the figures. Note scale differences between the subpanels.

Both the cold-water coral *Lophelia pertusa* and the sponge *Hymedesmia coriacea* incorporated algae and bacteria in all treatments (Fig 1). Mean tracer carbon (i.e. carbon from the isotopically-enriched food sources) incorporation rates by *L*. *pertusa* ranged from 0.08–0.17 μmol C_tracer_ mmol C_coral_^-1^ d^-1^ (Fig 1A) and tracer N incorporation rates ranged from 0.06–0.1 μmol N_tracer_ mmol N_coral_^-1^ d^-1^ (Fig 1D). For *H*. *coriacea*, tracer C incorporation ranged from 1.9–6.2 μmol C_tracer_ mmol C_sponge_^-1^ d^-1^ (Fig 1B) and tracer N incorporation rates ranged from 0.54–5.3 μmol N_tracer_ mmol N_sponge_^-1^ d^-1^ (Fig 1D). The tracer incorporation rates of the coral and the sponge were not significantly different between treatments (coral: *F*_1,19_ = 2.87, *P* = 0.11, sponge: *F*_1,19_ = 1.53, *P* = 0.23), food sources (coral: *F*_1,19_ = 0.35, *P* = 0.56, sponge: *F*_1,19_ = 3.19, *P* = 0.09) and elements (coral: *F*_1,19_ = 2.55, *P* = 0.13, sponge: *F*_1,19_ = 0.57, *P* = 0.46) nor was the interaction between treatment and food source (coral: *F*_1,19_ = 2.83, *P* = 0.11, sponge: *F*_1,19_ = 0.52, *P* = 0.48). However, the incorporation rates of the sponge were significantly higher than those of the coral in the ‘single species’ experiments (C: *F*_1,9_ = 101, *P* < 0.001, N: *F*_1,9_ = 106, *P* < 0.001). The C_tracer_ incorporation rates did not differ between food sources (*F*_1,9_ = 3.14, *P* = 0.11), while the N_tracer_ incorporation rates did differ significantly between food sources (*F*_1,9_ = 12.7, *P* < 0.01).

**Fig 2:**
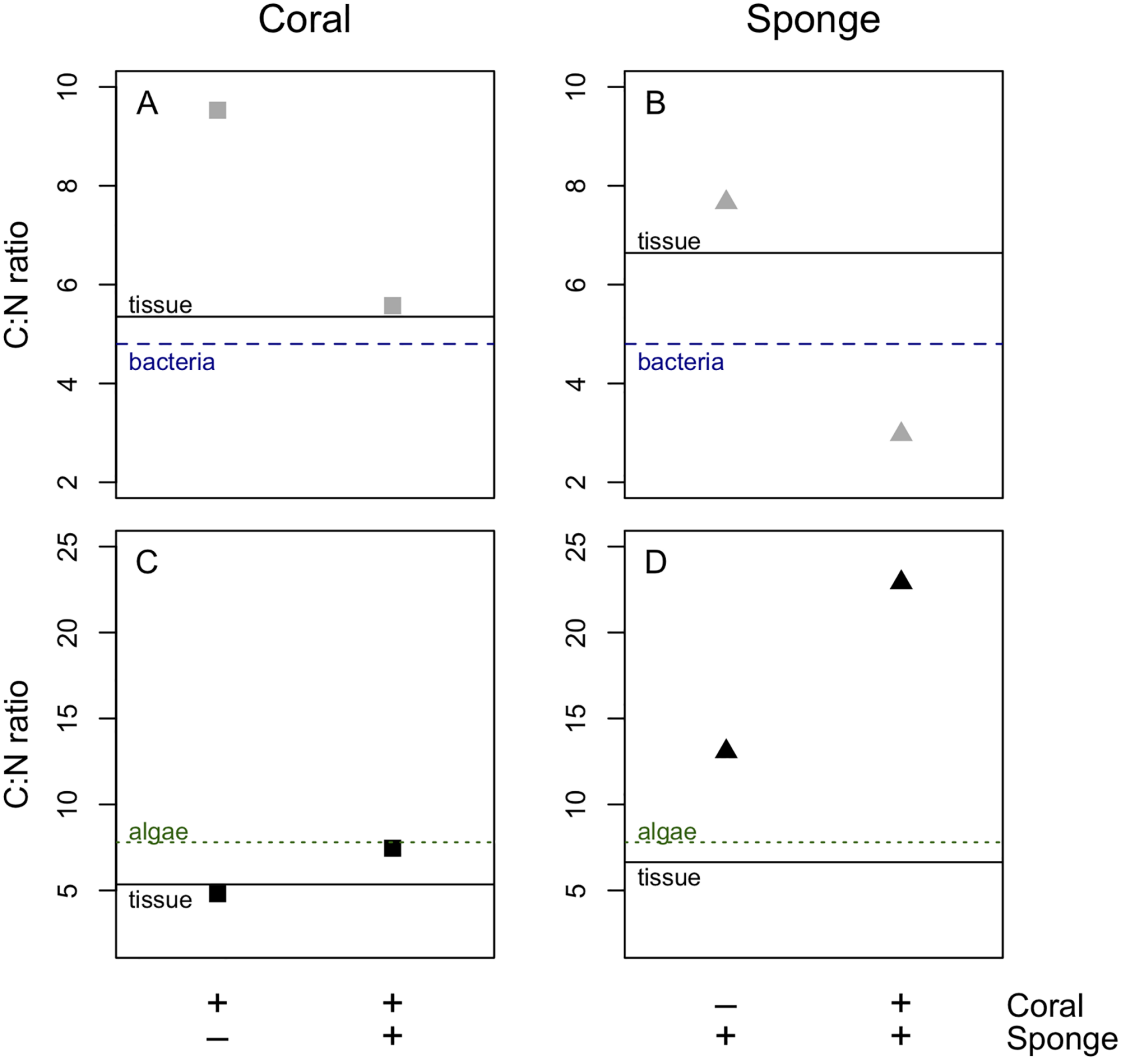
Stoichiometry of food incorporation. A) Mean C:N ratio of bulk coral tissue (solid line), bacterial food source (dashed line) and assimilation of bacteria into coral tissue (grey squares), B) mean C:N ratio of bulk sponge tissue (solid line), bacterial food source (dashed line) and assimilation of bacteria into sponge tissue (grey triangles), C) mean C:N ratio of bulk coral tissue (solid line), algal food source (dashed line) and assimilation of algae into the coral tissue (black squares) and D) mean C:N ratio of bulk sponge tissue (solid line), algal food source (dashed line) and assimilation of algae into sponge tissue (black triangles). The presence (‘+’) or absence (‘-‘) of a species is indicated below the figures. Note scale differences between subpanels A and B versus C and D.

It is not straightforward to evaluate the stoichiometry of resource uptake, as the uptake of C and N from algae (and bacteria) was measured in different incubations and thereby on different coral and sponge specimens (Table 1). Therefore, we calculated the stoichiometric uptake based on average C- and N-incorporation rates of both food sources and consider that only broad-scale differences will become apparent using this approach. The stoichiometric uptake of the coral *L*. *pertusa* is broadly in agreement with the C:N ratio of its tissue and the food sources, although there seems to be a preferential uptake of bacterial C in the coral only treatment (Fig 2A and 2C). The stoichiometric incorporation of bacteria by the sponge was broadly in agreement with the sponge tissue and the resource (Fig 2B). However, the sponge seemed to preferentially incorporate C from the algae in the ‘sponge only’ and ‘coral + sponge’ treatments (Fig 2D).

### Coral calcification

**Fig 3:**
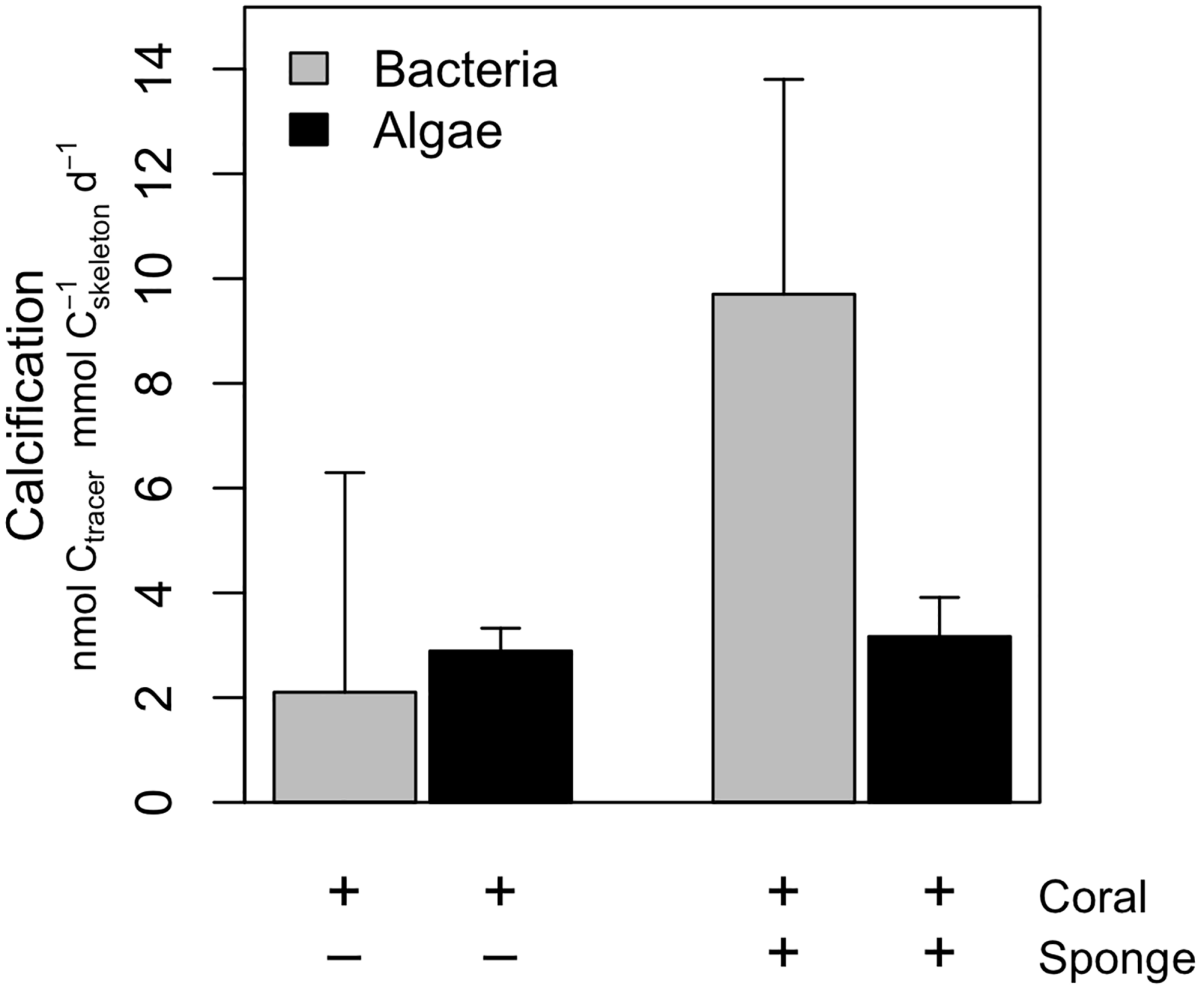
Coral calcification. Calcification rates expressed as nmol C_tracer_ mmol C_skeleton_^-1^ d^-1^ of the cold-water coral *Lophelia pertusa* when fed ^13^C-enriched bacteria or ^13^C-enriched algae in the presence (‘+’) or absence (‘-‘) of sponges as indicated below the figure.

The ^13^C tracer from algal and bacterial food sources was also traced back in the calcium carbonate skeleton of the coral (Fig 3). Calcification rates of the two food sources and treatments were in the same order of magnitude and ranged from 2.1–9.7 nmol C_tracer_ mmol C_skeleton_^-1^ d^-1^ (note the nmol scale for tracer). Calcification rates did not differ significantly between treatments (*F*_1,8_ = 3.0, *P* = 0.12) or food source (*F*_1,8_ = 2.7, *P* = 0.14).

### Respiration

**Fig 4:**
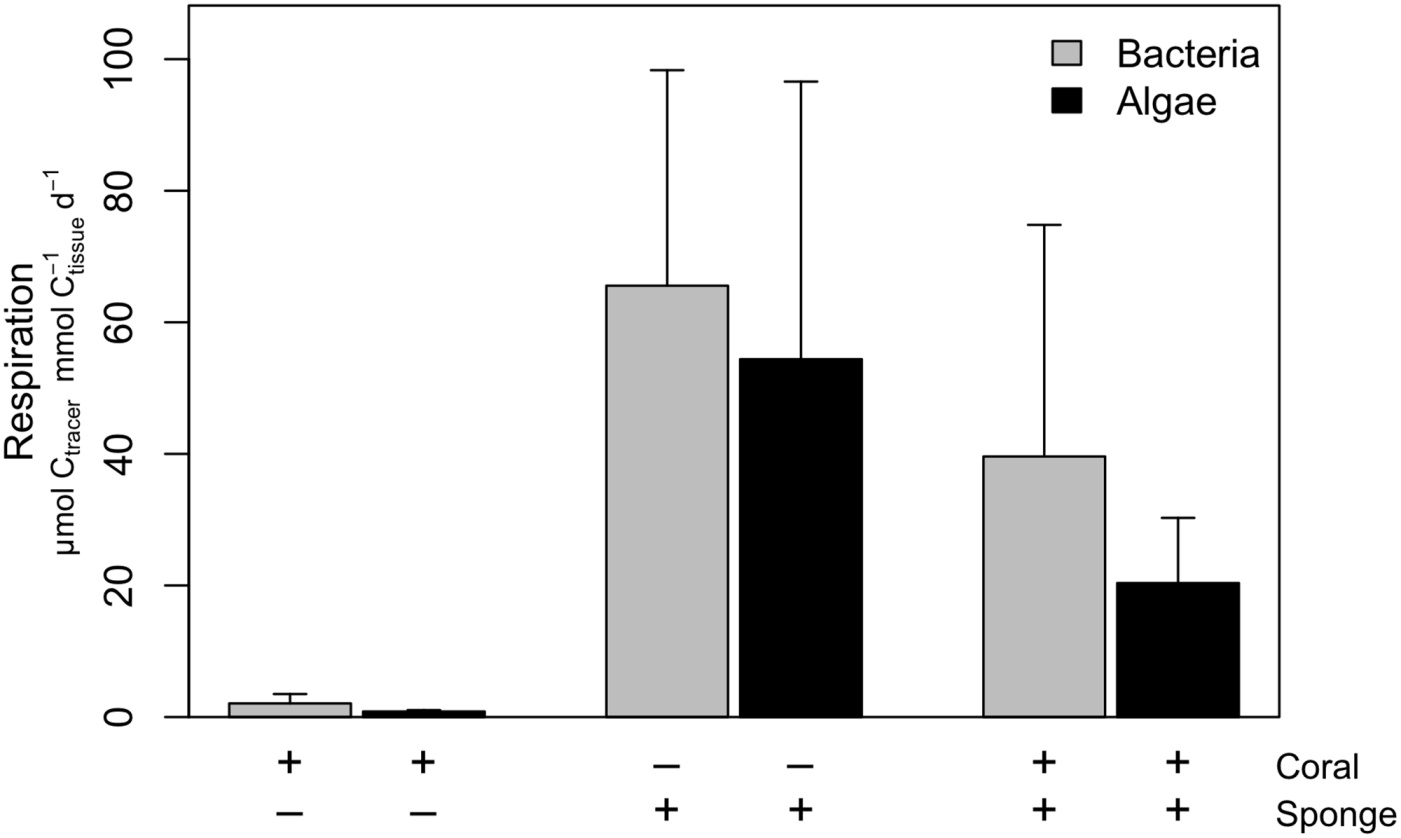
Respiration rates. Respiration rates expressed as μmol C_tracer_ mmol C_tissue_^-1^ d^-1^ of the incubations fed with ^13^C-enriched bacteria and ^13^C-enriched algae with only corals, with only sponges and with both taxa. The presence (‘+’) or absence (‘-‘) of a species is indicated below the figure.

The closed incubation after the 10-d feeding period showed respiration of the offered food sources by the coral and sponge in all treatments (Fig 4). Coral respiration rates of the isotope-enriched food ranged from 0.84–2.1 μmol C_tracer_ mmol C_coral_^-1^ d^-1^ whereas sponge respiration rates ranged from 54–66 μmol C_tracer_ mmol C_sponge_^-1^ d^-1^. The respiration rates in the treatment with corals and sponges cannot be attributed to a single taxon, but the biomass-specific respiration of the coral-sponge ‘consortium’ was between the coral and sponge rates and ranged from 20–40 μmol C_tracer_ mmol C_coral+sponge_^-1^ d^-1^. Respiration rates differed significantly between the coral and sponge (*F*_1,9_ = 78.2, *P* < 0.001), but did not differ significantly between food sources (*F*_1,9_ = 2.3, *P* = 0.16).

### Carbon budget of the experiment

To estimate the fate of the added food sources during the experimental period, a carbon budget was calculated for the coral and sponge (based on the ‘single species only’ treatment data) results for incorporation, calcification and respiration under the assumption that these rates were constant over the 10-day feeding period. Note that the contribution of respiration in the budget was only determined at the end of the 10-day feeding period, while the incorporation rates were averaged over the whole period and that other loss processes are ignored (see Discussion below).

The total amount of carbon processed by the coral during the 10-d experiment (i.e. sum of incorporation, respiration and calcification) in the separate food treatments amounted to 273 μmol bacterial-C and 111 μmol algal-C, and for the sponge it was 345 μmol bacterial-C and 219 μmol algal-C (Table 2). For both taxa, the dominant fate was respiration. Of the total carbon added (2,708 μmol algal-C and 8,125 μmol bacterial-C), between 10 and 13% of the algal-C and between 0.014 and 0.027% of the added bacterial-C could be accounted for, while the remainder was not processed or could not be accounted for. The comparatively small differences in total processing rates between the two taxa in the experiment (Table 2) contrast with the clear differences in the processing rates presented in Figs 1 and 3, which is explained by the 29 ± 9 times (mean ± standard deviation) higher coral biomass in the incubations as compared to the sponge biomass.

**Table 2:** Carbon budget during the 10-d experiment. Total carbon budget (μmol C, mean ± sd) of the coral *Lophelia pertusa* and the sponge *Hymedesmia coriacea* during the 10-day experiment. The amount ‘Unaccounted’ was calculated as the added food minus the other terms.

		Coral	Sponge
Bacteria	Tissue incorporation	14.8 ± 1.7	37.2 ± 43.7
	Respiration	256.1 ± 101.8	308.2 ± 82.8
	Calcification	2.3 ± 4.5	
	Unaccounted	7,852 ± 105	7,777 ± 75
Algae	Tissue incorporation	9.4 ± 2.3	8.0 ± 4.8
	Respiration	98.5 ± 40.7	210.7 ± 157.0
	Calcification	3.1 ± 0.53	
	Unaccounted	2,597 ± 43	2,489 ± 161

### Net growth efficiency

**Fig 5:**
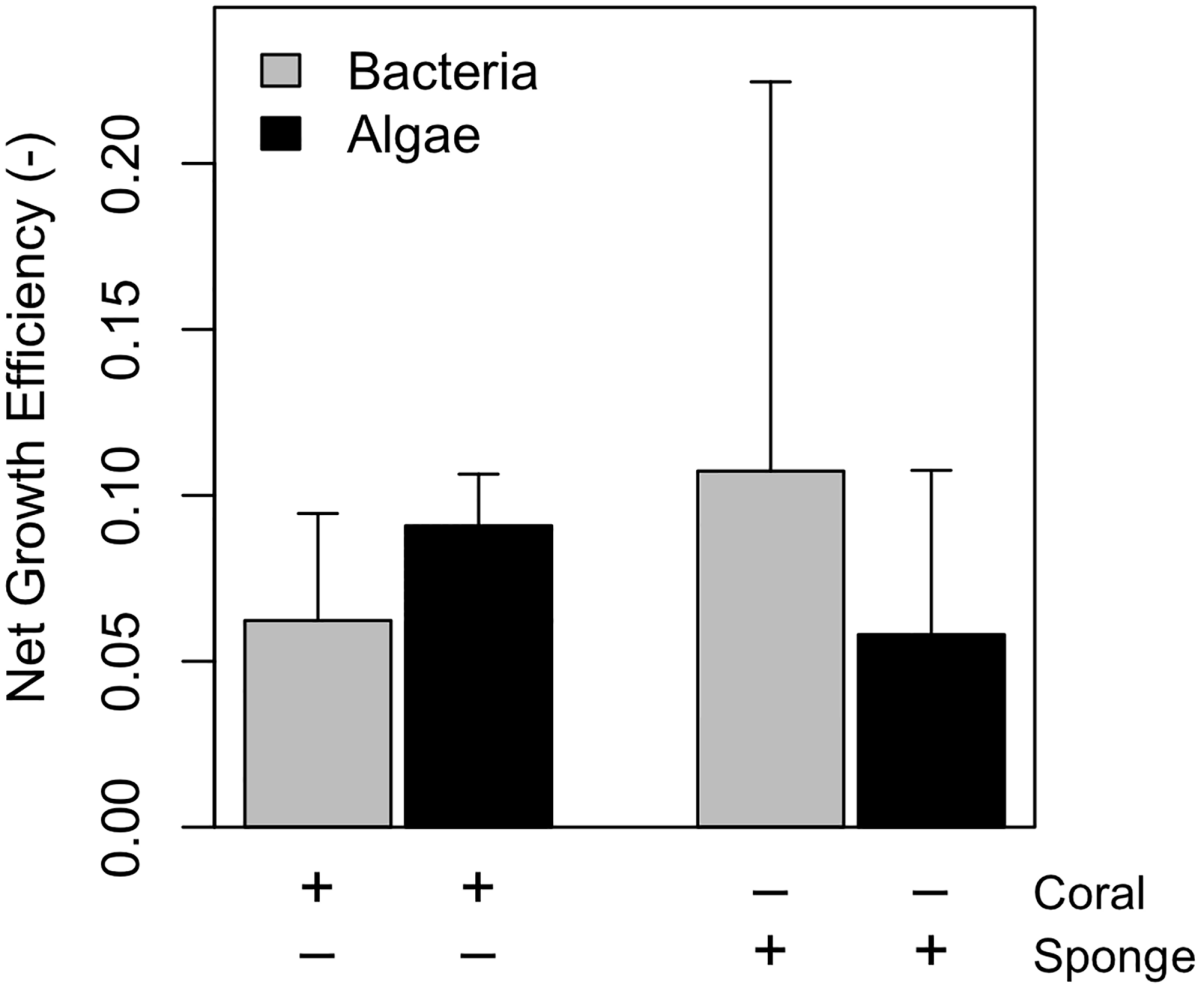
Net growth efficiency. The net growth efficiency (NGE) of the incubations fed with ^13^C-enriched bacteria and ^13^C-enriched algae with corals or sponges. The presence (‘+’) or absence (‘-‘) of a species is indicated below the figure.

The net growth efficiency ranged from 5.8 to 11.0% (Fig 5). The net growth efficiency did not differ significantly between species (*F*_1,9_ = 0.34, *P* = 0.57) and food source (*F*_1,9_ = 0.010, *P* = 0.92).

## Discussion

### Niche overlap between a coral and its associated sponge

The coral and the sponge both assimilated and respired the suspended bacteria and algae, indicating niche overlap between these suspension feeders. The incorporation rates were, however, not significantly different for the two food sources for both taxa, although bacteria were added in a threefold higher quantity as compared to the algae. While it was surprising to see that the mean incorporation and calcification rates of the coral when fed with bacteria were higher in the ‘coral + sponge’ treatment as opposed to the ‘coral only’, this difference was not significant. The coral *L*. *pertusa* is a known opportunistic suspension feeder [29], capable of feeding of dissolved organic matter [17, 18] and variously sized suspended particulates [17, 41–43].

The sponge *Hymedesmia coriacea* did not show a clear preferential feeding on bacteria as reported for other sponges [24, 44, 45]. Similar uptake of pico- (equaling bacteria) and nanoplankton (equaling algae)-derived carbon under comparable conditions has been observed for the temperate sponge *Mycale lingua* (Pile et al. 1996), which is found on CWC reefs [46]. Smaller-sized phytoplankton may therefore be an important diet contribution of CWC reef sponges. The algal cells are retained in the aquiferous canal system of the sponge and while particles <4 μm are preferentially retained in the filter cell, i.e. choanocytes, of the sponge, larger particles are captured by pinacocytes, epithelial cells [47]. Hence, a modulation of selective cell types might explain the differences in assimilation rates among species and allows niche segregation between suspension feeders on the CWC coral reef. Besides uptake of suspended particles, uptake of dissolved organic matter and dissolved inorganic carbon (i.e. chemoautotrophy by symbiotic microbes) have been observed for CWC reef sponges [48, 49].

Niche overlap between taxa may also result in exploitative competition for suspended resources. We did not find experimental evidence for exploitative competition as the co-occurrence of a coral and its associated sponge did not significantly reduce incorporation rates. However, exploitative competition is only expected when resource concentrations are limiting. The provided food concentrations during the incubations (108 μmol C L^-1^) were substantially higher as compared to natural conditions where organic carbon concentrations of 5.8 μmol C L^-1^ [Røst reef, 50], 3.6 to 8.9 [Tisler reef, 33] and 0.75 to 10.8 μmol C L^-1^ (several North Atlantic reef locations [several Atlantic reefs, 51] have been measured. Excess feeding was decided for to ensure uptake of measurable food quantities by the investigated species. As a result, only a small fraction of the resources that were fed during this study could be traced back in tissue, skeleton (only for coral) or in the dissolved inorganic carbon pool following respiration. Although this budget ignores release of ^13^C/^15^N tracer by corals in the form of secondary metabolites or dissolved organic matter [50], and the release of cellular debris and undigested food [49], it is evident that most labelled food was not consumed during the daily feeding period. Food availability at cold-water coral reefs is however strongly variable throughout the year [14, 19, 52] and we infer that, if exploitative competition between corals and sponge occurs in nature, it will be during periods of low food concentration. Incubations with sponges and corals under natural concentrations of organic matter may confirm whether niche overlap will induce exploitative competition.

### Species-specific differences in food incorporation

Our experiments did show a highly significant ‘species effect’, with incorporation and respiration rates of the isotopically-enriched food sources being more than an order of magnitude higher for the sponge as compared to the coral. We here discuss two, non-mutually exclusive, explanations for this striking difference.

The first explanation is that the higher uptake and processing of the isotopically-enriched food by the sponge is due to a higher natural metabolic rate as compared to the coral. We cannot verify this from the experiment as we unfortunately have no metabolic rate data such as oxygen consumption. We are unaware of metabolic rate data for *H*. *coriacea* from the literature. However, a compilation of respiration rate data from organisms from a cold-water coral reef at Rockall Bank and literature data cold-water sponges, suggests that the metabolic rate of cold-water sponges are slightly higher (range of 0.001–0.006 d^-1^) than that of the cold-water coral *L*. *pertusa* (i.e. 0.0015 d^-1^) [53]. Additional metabolic rate data are obviously needed to verify this, but if true, sponges would have a disproportionately higher role in the metabolism of a cold-water coral ecosystem. Accurate biomass estimations of corals and sponges on natural cold-water coral reefs are needed to assess their role in the cycling of organic carbon within these ecosystems.

The second explanation for the different processing rates of the isotopically-enriched food sources may be related to the feeding mode of these sessile species. The coral *L*. *pertusa* is a passive suspension feeder that captures particles from the water column following physical contact with nematocyst-laden tentacles [29, 54, 55], while the demosponge *H*. *coriacea* is an active suspension feeder that uses flagellated cells to pump ambient water through its filtration system to capture suspended (and dissolved) resources [56, 57]. Active suspension feeders are hypothesized to feed more effectively on smaller particles, while passive feeders are better at retaining larger, more energy-rich particles [58]. In our experiment, however, roughly equally-sized food sources were used and no differences in incorporation rates for algae and bacteria were observed for both species. Another key difference between these two feeding modes is that the particulate food *flux* is an important factor affecting the food uptake of passive suspension feeders as it increases encounter rates between particles and feeding appendages, whereas particulate food *concentration* is more relevant for active suspension feeders as this increases uptake rate at a given pumping activity [59]. As an example, Sebens [60] found that the colony size of the passive suspension feeder *Alcyonium siderium* (Linnaeus, 1758) was higher at more exposed sites as compared to a site with hydrodynamic calmer conditions, despite having similar seston concentration. Sponges may take advantage of current-induced flow to enhance feeding [61], a mechanism that would blur this generalization. Recent evidence for glass sponges however indicates that passive flow is energetically favorable only for porous, thin-walled sponges living in high flow environments [56]. We therefore argue that the difference in feeding mode may partly explain the observed difference in uptake of labelled resources, as the experimental setup (i.e. a circular incubation chamber) apparently favored the active suspension feeding modus of the sponge. Experiments in which flux and concentration are varied in hydrodynamic controlled conditions, e.g., flumes, are needed to verify this, but if found valid, this may set feeding constraints on suitable habitats for cold-water corals and sponges in nature.

The energetic costs involved in pumping activity have been considered to be negligible [<4% of the daily energy expenditure, 62], but recent estimates of pumping activity by glass sponges suggest that the head loss over the filter system was previously underestimated with a factor 10, warranting an upward revision of the pumping costs in the energy budget to almost 30% [56]. Costs for active sponge pumping did however not translate into lower net growth efficiency for the sponge as compared to the coral.

In conclusion, the cold-water coral and its associated sponge both utilized the suspended bacteria and algae as food source indicating niche overlap between these taxa. A clearly higher food assimilation and respiration was found for the sponge as compared to the cold-water coral. We hypothesize that the active suspension feeding mode of the sponge explains the observed differences in resource uptake as opposed to the passive suspension feeding mode of the cold-water coral. These feeding mode differences may set constraints on suitable habitats for cold-water corals and sponges in nature.
